# Transcriptomic responses of *Aspergillus flavus* to temperature and oxidative stresses during aflatoxin production

**DOI:** 10.1038/s41598-021-82488-7

**Published:** 2021-02-02

**Authors:** Fei Tian, Sang Yoo Lee, So Young Woo, Hwa Young Choi, Seongeun Heo, Gyoungju Nah, Hyang Sook Chun

**Affiliations:** 1 Food Toxicology Laboratory, School of Food Science and Technology, Chung-Ang University, Anseong, Korea; 2 Genome Analysis Center at National Instrumentation Center for Environmental Management, Seoul National University, Seoul, Korea

**Keywords:** Fungal genomics, Chemical biology

## Abstract

Aflatoxin is a group of polyketide-derived carcinogenic and mutagenic secondary metabolites produced by *Aspergillus flavus* that negatively impact global food security and threaten the health of both humans and livestock. Aflatoxin biosynthesis is strongly affected by the fungal developmental stage, cultivation conditions, and environmental stress. In this study, a novel float culture method was used to examine the direct responses of the *A. flavus* transcriptome to temperature stress, oxidative stress, and their dual effects during the aflatoxin production stage. The transcriptomic response of *A. flavus* illustrated that the co-regulation of different secondary metabolic pathways likely contributes to maintaining cellular homeostasis and promoting cell survival under stress conditions. In particular, aflatoxin biosynthetic gene expression was downregulated, while genes encoding secondary metabolites with antioxidant properties, such as kojic acid and imizoquins, were upregulated under stress conditions. Multiple mitochondrial function-related genes, including those encoding NADH:ubiquinone oxidoreductase, ubiquinol-cytochrome C reductase, and alternative oxidase, were differentially expressed. These data can provide insights into the important mechanisms through which secondary metabolism in *A. flavus* is co-regulated and facilitate the deployment of various approaches for the effective control and prevention of aflatoxin contamination in food crops.

## Introduction

Aflatoxin is a group of polyketide-derived secondary metabolites produced by the facultative plant parasite *Aspergillus flavus*. Aflatoxin is well recognized for its potent toxicity, mutagenicity, teratogenicity, and carcinogenicity and is classified as a group 1 carcinogen by the International Agency for Research on Cancer (IARC). Aflatoxin contamination is a worldwide food safety concern and impacts both the marketability and safety of multiple food crops^1^. Most aflatoxin-related losses are primarily to the market rather than to human or animal health. Economic losses due to aflatoxin contamination are estimated at hundreds of millions of dollars globally, with maize and peanuts being the most seriously affected food crops^2^. Meanwhile, climate change have been predicted to significantly affect the infection of food crops by mycotoxigenic fungi and mycotoxin contamination, which will bring new challenges to the security of global staple food commodities^3^.

Climate change-induced environmental stresses, such as drought and temperature and oxidative stresses, can exacerbate aflatoxin contamination in food crops^4^. Over the last decade, numerous research efforts have been undertaken to understand how environmental stress affects aflatoxigenic fungi and aflatoxin biosynthesis^5–8^. Although the function of aflatoxin production in fungal biology is still unclear, there is clear evidence that aflatoxin biosynthesis is regulated by oxidative stress. Oxidative stress can be induced by either temperature changes or reactive oxygen species (ROS), which are generated by host cells in oxidative bursts that occur during plant-pathogen interactions^9–11^. Even though both temperature change and oxidative stress are closely connected with ROS metabolism in fungal cell, the effects of temperature change-induced stress and extracellular ROS-induced oxidative stress on fungal cells have never been compared. Given that ROS play important roles in the regulation of both fungal pathogenesis and secondary metabolism^12,13^, a deep analysis of the responses of *A. flavus* to temperature stress, oxidative stress, and a combination of the two may further improve our understanding of fungal pathogenesis and the regulation of aflatoxin production in this fungus, thereby aiding efforts to prevent aflatoxin contamination under climate change.

In addition to environmental stress, fungal metabolism is also strongly affected by both the developmental stage and the cultivation conditions of the fungus. In solid substances, such as plant tissues and food products, fungi are usually immobilized and develop as colonies either on the surface or embedded within the solid material. However, studies on fungal growth and metabolism are typically conducted in broth medium, and data obtained from liquid cultures may not accurately describe the metabolic conditions occurring in immobilized growth forms^14,15^. Meanwhile, fungal responses to environmental stimuli are highly dependent on the stage of growth^16^. Indeed, fungal cells have a complex “temporal switch” that regulates activation of genes involved in secondary metabolic pathways and controls the flow of primary metabolites through them. This results in maximal secondary metabolite production at specific time points in the fungal life cycle^17^. In laboratory conditions, aflatoxin production in *A. flavus* usually begins after 24–48 h of incubation in induction medium, at which time spore germination is complete, and tends to stop when fungal growth reaches stationary phase. Therefore, it is expected that *A. flavus* may exhibit different responses to environmental stress at different growth phases.

In the present study, in order to improve our understanding of aflatoxin biosynthesis regulation in *A. flavus*, we performed a global gene expression analysis on *A. flavus* at its aflatoxin production stage using a novel float culture method^18^. Genes that specifically responded to higher temperature-induced temperature stress, extracellular ROS (H_2_O_2_)-induced oxidative stress, or the dual effects of temperature and oxidative stresses were identified. Gene ontology (GO) enrichment revealed the close relationship between secondary metabolic processes and environmental stress responses. The response of mitochondrial function-related genes was also examined. These data can help us to better understand the response of *A. flavus* to environmental stress, provide insights into important mechanisms related to the regulation of its secondary metabolic systems, and facilitate the deployment of various approaches for the effective control and prevention of aflatoxin contamination in food crops.

## Results

### Transcriptome sequencing

**Figure 1:**
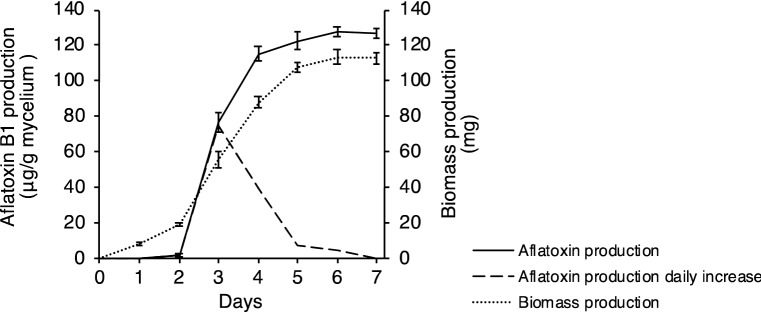
Aflatoxin production and biomass production of *A. flavus* KCCM60330 in float cultures.

**Figure 2:**
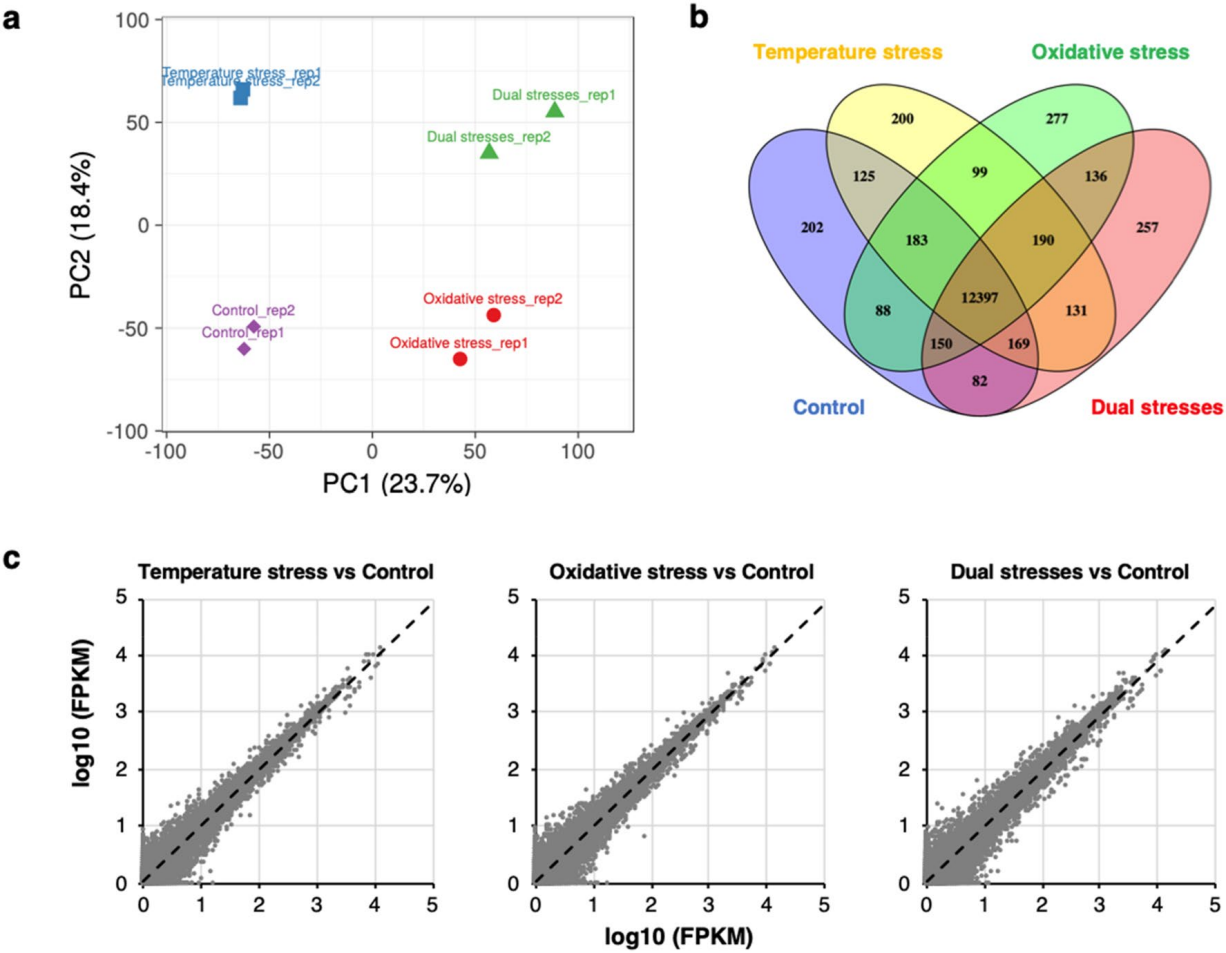
Overview of RNAseq results. (**a**) Principal component analysis of the gene expression profiles in different treatment groups (violet diamond control group; blue square temperature stress group; red circle oxidative stress group; green triangle dual stresses group) was conducted using ClustVis (https://biit.cs.ut.ee/clustvis/). (**b**) Venn diagram of genes expressed in different treatment groups (FPKM ≥ 2) generated with Venny 2.1.0 software. (**c**) Pairwise comparison of the overall levels of gene expression under control and stress conditions.

To examine the transcriptomic responses of *A. flavus* to temperature stress and oxidative stress during aflatoxin production (48 to 72 h after inoculation, Fig. 1), we cultured the aflatoxigenic reference strain KCCM60330 using a novel float culture method^18^. All the cultures were incubated at 28 °C for 48 h, then subjected to a higher temperature (33 °C) and/or oxidative stress that was induced by H_2_O_2_ (5 mM) for an additional 24 h. Transcriptomes were sequenced on an Illumina Hi-seq 2500 system, and differences in the expression profiles among the different treatments were analysed. In total, 178.9 million quality-filtered reads, corresponding to 54,026,617,670 nucleotides, were generated from eight samples with an average of 22.4 million reads per sample. A total of 23,966 contigs were assembled through genome-guided transcriptome assembly using Trinity assembler^19^ with a size range of 301 to 23,487 bp (Table 1). After removing isoforms, 19,527 unigenes were identified (Supplementary Data S1). Principal component analysis showed that the expression profiles of *A. flavus* under temperature or oxidative stress were clearly segregated, suggesting that both stresses significantly altered the expression profiles of *A. flavus* (Fig. 2a). Among them, 14,686 genes were found to possess values for fragments per kilobase of exon per million fragments mapped (FPKM) ≥ 2 (expressed genes) under at least one treatment condition. Totally, 12,397 (84.41%) genes were found to be expressed in all four treatment groups. Meanwhile, 202 (1.38%) and 200 (1.36%) genes were found to be uniquely expressed in the control group and temperature stress group, respectively. In addition, 277 (1.89%) genes were expressed exclusively in the oxidative stress group, whereas 257 (1.75%) genes were expressed exclusively in the dual stresses (temperature stress + oxidative stress) group (Fig. 2b, Supplementary Data S1). A pairwise comparison between samples from the control and stress conditions showed that the levels of overall gene expression in the treatments were highly similar to those in the control (Fig. 2c). Unigenes were annotated by aligning their sequences against those from the uniprot database, the *A. flavus* coding sequences (CDS) data, and the NCBI non-redundant (nr) protein database using an e-value threshold of 10^–5^. A total of 7123 (36.5%), 13,498 (69.1%), and 15,558 (76.7%) unigenes were annotated, respectively. Moreover, several previously uncharacterized genes were also found to exhibit high expression levels (FPKM > 200) in all tested samples, and many of them were differentially expressed under stress conditions (Supplementary Data S1).

**Table 1:** Summary of genome-guided Trinity transcriptome assembly results.

Category	Value
Total length (bp)	26,832,353
Number of contigs	23,966
Average length (bp)	1119.6
Maximum length (bp)	23,487
Minimum length (bp)	301
N75 length (bp)	1036
N50 length (bp)	1632
N25 length (bp)	2384
GC content (%)	50.75

### *A. flavus* responses to temperature stress and oxidative stress during aflatoxin production

**Figure 3:**
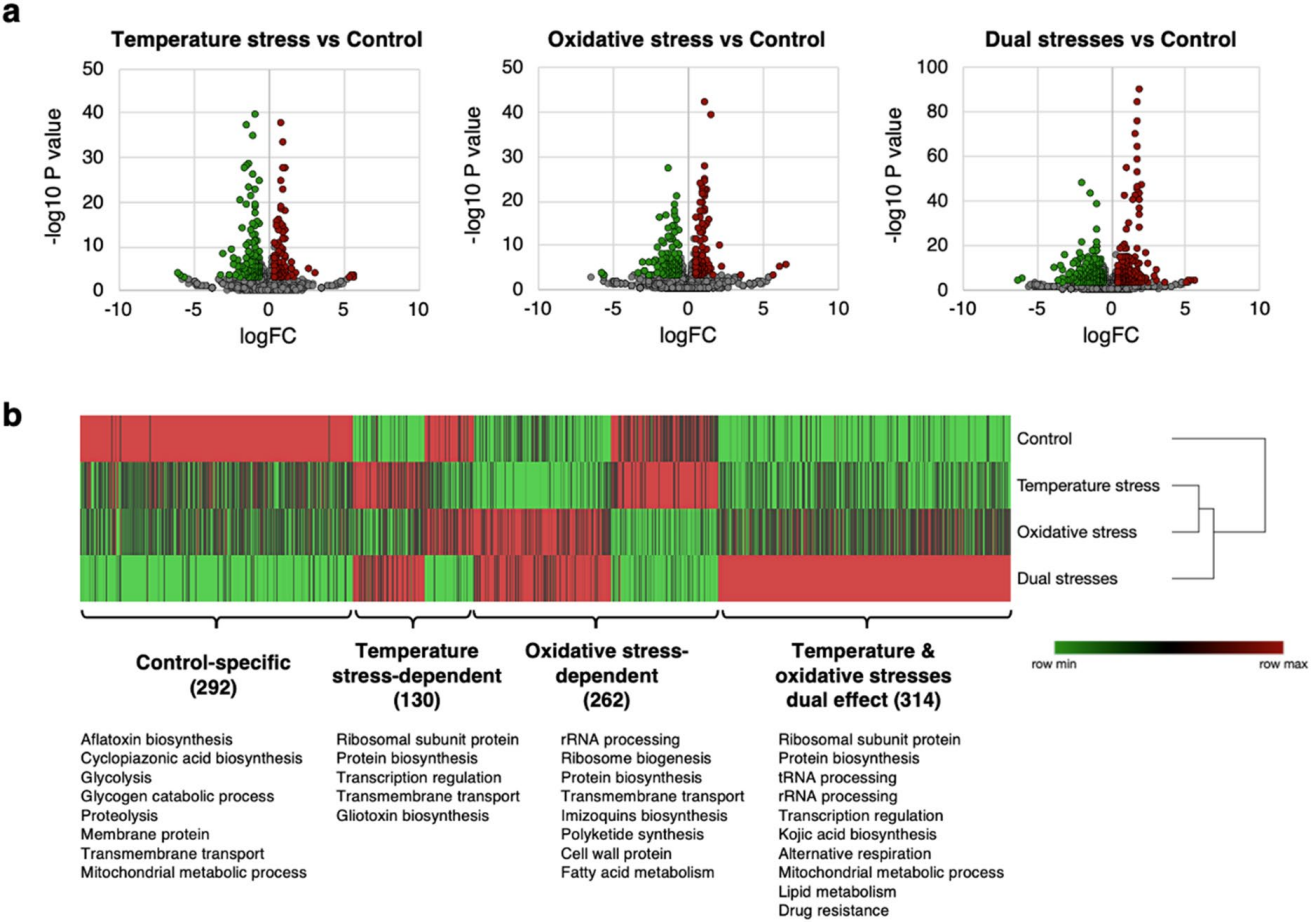
Differential gene expression analysis. (**a**) Volcano plot of detected genes indicating DEGs under different stress conditions that were significantly upregulated (red circle), significantly downregulated (green circle), or nonsignificant (grey circle) (False Discovery Rate (FDR) < 0.05). (**b**) Heatmap indicates relative expression of DEGs that specifically responded to temperature stress, oxidative stress, and the dual stresses treatment. DEGs that exhibited significantly higher expression levels under control condition than under stress conditions are described as “Control-specific”. Genes that were differentially expressed under temperature stress but not under oxidative stress are described as “Temperature stress-dependent”. Genes expressed differentially under oxidative stress but not under temperature stress are described as “Oxidative stress-dependent”. Genes that exhibited significantly higher expression levels under the dual stresses treatment are labelled “Temperature & oxidative stresses dual effect”. The number of DEGs included in each of these categories is indicated. The biological functions (containing 2 to 39 DEGs) represented in each category are listed below their respective category labels.

In order to compare the transcriptional responses of *A. flavus* to temperature stress and oxidative stress, we first identified the differentially expressed genes (DEGs) among the treatment groups (Fig. 3a). A total of 239 and 266 DEGs were differentially expressed under temperature stress and oxidative stress, respectively, relative to the control, whereas 635 DEGs were detected in the dual stresses group. Many of the identified DEGs were found to be expressed at significantly higher levels in the control than under the stress conditions (the control-specific DEGs, Fig. 3b), including up to 26 aflatoxin biosynthetic genes and multiple genes involved in glycolysis, proteolysis, and mitochondrial functions (Fig. 3b, Supplementary Data S2). Genes differentially expressed specifically under temperature stress but not oxidative stress (the temperature stress-dependent DEGs, Fig. 3b) and those differentially expressed under oxidative stress but not temperature stress (oxidative stress-dependent DEGs, Fig. 3b) were identified. The major biological functions of these DEGs are summarized in Fig. 3b. According to the results, the dual stresses treatment had a markedly stronger effect on gene expression collectively than either temperature stress or oxidative stress alone. Multiple previously uncharacterized genes were also identified as DEGs under temperature stress, oxidative stress, and their combination. Meanwhile, functional categories (GO) analysis showed that temperature stress and oxidative stress exhibit similar effects on secondary metabolism and mycotoxin biosynthesis, but oxidative stress had a larger influence on proteolysis and cellular lipid metabolism (Supplementary Data S3). The dual stresses treatment affected more functional categories than either single stress treatment, and these categories included ion, cation, and nitrogen transmembrane transport.

### Temperature and oxidative stresses downregulate aflatoxin biosynthetic gene expression and interrupt related metabolism pathways

**Figure 4:**
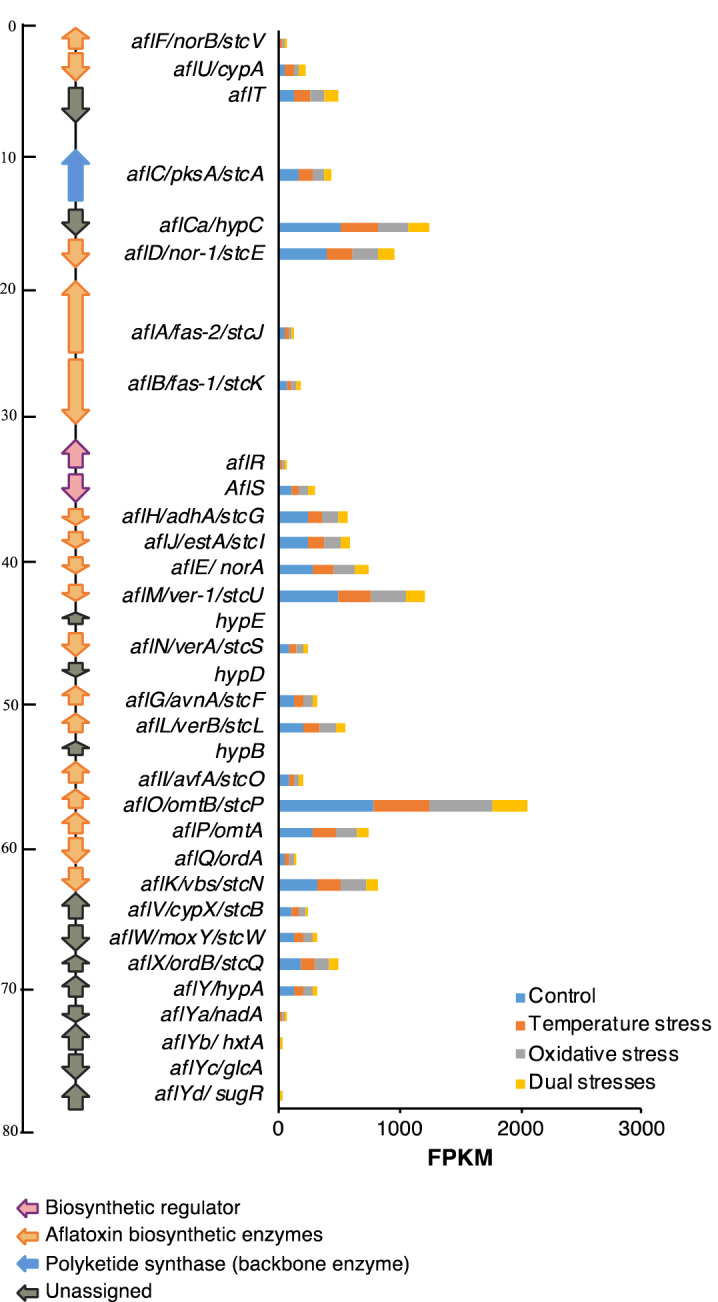
Relative expression of aflatoxin biosynthetic genes. Bars indicate the expression (FPKM) of genes under different treatment conditions. The relative locations of each gene in the aflatoxin biosynthetic gene cluster are shown on the left side.

According to the results, most genes involved in the biosynthesis of aflatoxin were downregulated in response to temperature stress and oxidative stress. The most significant changes in expression mainly came from the genes encoding enzymes involved in oxidation–reduction reactions and reactive oxygen species (ROS) metabolism, including monooxygenases (*aflG*, *aflI*, *aflV*, *aflN*, *aflW*, *aflX*), reductases (*aflD* and *aflE*), and dehydrogenases (*aflH* and *aflM*), which exhibited inhibition rates of 36.45% ~ 71.40% (Fig. 4, Supplementary Table S1). The expression of genes encoding the internal regulators, *aflR* and *aflS*, was reduced by > 27.11% in samples treated with temperature stress or oxidative stress, and > 44.31% in samples treated with dual stresses. The gene encoding polyketide synthase (*aflC*), the backbone enzyme of this cluster, also exhibited 37.04% and 48.77% inhibition under temperature stress and oxidative stress, respectively, and when temperature and oxidative stresses were both applied, its expression was reduced by 68.64%. The reductions of aflatoxin production in float cultures after treatment with temperature and oxidative stresses were also detected (Supplementary Table S2). The reduction of aflatoxin production under temperature stress (53.2%) was greater than that under oxidative stress (24.5%). The dual stresses treatment resulted in the strongest reduction (66.5%). The reductions in the expression of *aflR*, *aflS*, and *aflD*, together with that of 9 other genes involved in different biological functions, were validated using quantitative reverse transcription PCR (qRT-PCR) analysis, and similar gene expression trends were observed (Supplementary Fig. S1). An overall correlation value of 0.86 between RNAseq and qRT-PCR was observed for all selected genes, which indicates that there was substantial agreement between the RNAseq and qRT-PCR results.

**Figure 5:**
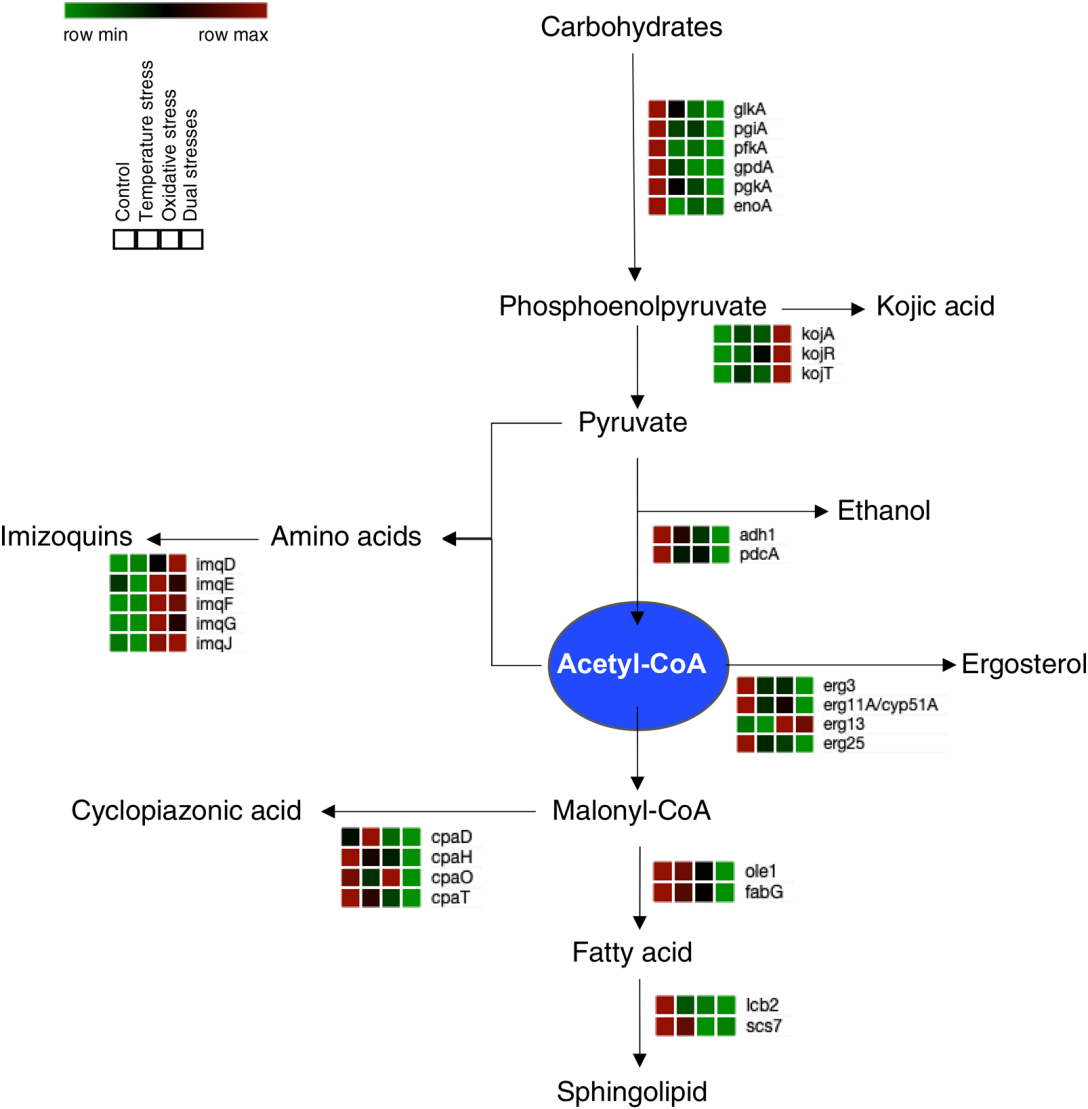
Differential expression of genes involved in glycolysis and secondary metabolism. Tables beside the gene annotations indicate relative gene expression in samples from the control, temperature stress, oxidative stress, and dual stresses groups, respectively.

Furthermore, genes encoding glycolysis enzymes, such as glucokinase (EC 2.7. 1.2), glucose-6-phosphate isomerase (EC 5.3.1.9), 6-phosphofructokinase (EC 2.7.1.11), glyceraldehyde 3-phosphate dehydrogenase (EC 1.2.1.12), phosphoglycerate kinase (EC 2.7.2.3), and enolase (EC 4.2.1.11), were found to be downregulated by both temperature stress and oxidative stress (Fig. 5). In addition to these, many genes connected to the metabolism of acetyl-CoA, the major building block of aflatoxin, were also differentially expressed. Cyclopiazonic acid is an indole-tetramic acid neurotoxin produced by strains of *Aspergillus* and *Penicillium* for which acetyl-CoA is also used as a building block. Members of the cyclopiazonic acid biosynthetic pathway (*cpaD*, *cpaH*, *cpaO*, *cpaT*) were found to be downregulated in stress-treated samples in a manner similar to that observed for the aflatoxin biosynthetic genes. Expression of the genes encoding acyl-CoA desaturase and ceramide very long chain fatty acid hydroxylase, both of which are enzymes involved in lipid metabolism, was downregulated by oxidative stress. Among the genes involved in ergosterol biosynthesis, *erg3*, *erg11*, and *erg25* were downregulated by both temperature and oxidative stress, whereas *erg13* expression was upregulated by oxidative stress. Moreover, genes involved in the biosynthesis of imizoquins (*imqD*, *imqE*, *imqF*, *imqG*, *imqJ*), a group of tripeptide-derived alkaloids that provide protection against oxidative stress, were upregulated by oxidative stress regardless of the presence or absence of temperature stress. Another secondary metabolite involved in fungal oxidative stress response is kojic acid. Genes encoding enzymes in the kojic acid biosynthetic pathway (*kojA*, *kojR*, *kojT*) were also found to be upregulated by the combined effects of temperature and oxidative stress. In particular, *kojA* was expressed at a much higher level (FPKM > 2500) than the other secondary metabolic genes.

### Expression of stress responsive upstream regulators

Several upstream regulators of secondary metabolite production and environmental stress responses were also found to be differentially expressed in the stress treatments relative to the control. The bZIP transcription factors *atfA* and *atfB*, which regulate aflatoxin production in response to oxidative stress, were downregulated by both temperature stress and oxidative stress. Another *ap-1*-like bZIP transcription factor (AFL2T_09270) was upregulated under stress conditions. A *skn7-*like (AFL2T_02624) transcription factor was also upregulated in response to both temperature stress and oxidative stress. Among the velvet protein family, the genes encoding LaeA and VeA were found to be upregulated by oxidative stress and temperature stress, respectively. The VelB-encoding gene exhibited decreased expression under the stress treatments, while the stress treatments increased expression of the VelC-encoding gene. The central regulatory genes for asexual sporulation, *brlA*, *abaA*, and *wetA*, were also examined. However, they showed no significant response to either temperature stress or oxidative stress. Several previously uncharacterized transcription factors were also found to be expressed under both the control and stress conditions (Supplementary Data S4).

### Temperature and oxidative stress affect mitochondrial function-related genes

**Figure 6:**
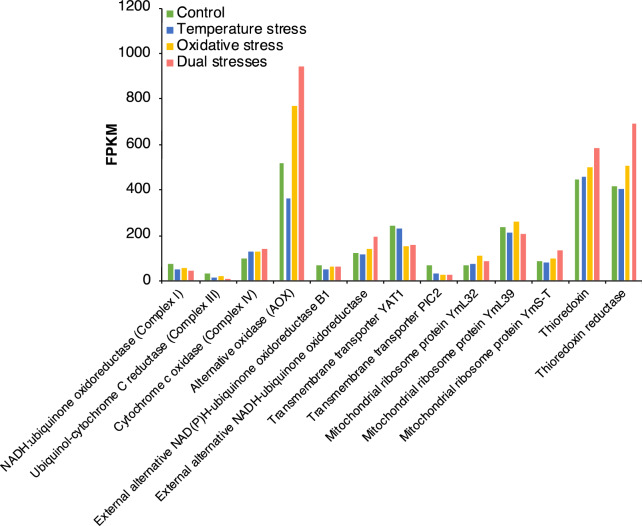
Expression of selected genes encoding mitochondrial function-related enzymes. Bars indicate the expression (FPKM) of genes under different treatment conditions.

Genes encoding mitochondrial respiration enzymes, including the NADH:ubiquinone oxidoreductase (mitochondrial complex I, EC 7.1.1.2), the ubiquinol-cytochrome C reductase (mitochondrial complex III, E.C. 1.10.2.2), and the cytochrome C oxidase (mitochondrial complex IV, EC 1.9. 3.1), were found to be affected by both temperature stress and oxidative stress (Fig. 6). Genes encoding alternative oxidase (AOX) and the external alternative NADH-ubiquinone oxidoreductase, which are members of the alternative respiration pathway, were both upregulated in response to oxidative stress. AOX-encoding genes were expressed at relatively higher levels (FPKM > 350) and were significantly regulated by both temperature stress and oxidative stress. Several genes encoding mitochondrial membrane transporters were also affected by the stress treatments. The gene *yat1*, which encodes a mitochondrial carnitine O-acetyltransferase that transfers acetyl-CoA into mitochondria, was downregulated in response to oxidative stress regardless of the presence or absence of temperature stress. The mitochondrial phosphate carrier protein PIC2 was also found to be downregulated in stress-treated samples. Several genes encoding mitochondrial ribosome proteins, including YmL32, YmL39 and YmS-T, were also regulated by both temperature stress and oxidative stress. The genes encoding thioredoxin and thioredoxin reductase were both expressed at relatively higher levels and were significantly upregulated by the dual stresses treatment.

## Discussion

Environmental factors-induced abiotic stresses have complex influences on fungal secondary metabolism. Several studies have shown that mild abiotic stress and acute abiotic stress seem to affect mycotoxin biosynthesis differently^20,21^. When compared with acute stress, mild stress is more commonly being induced in the natural environment. Meanwhile, it has been reported that mildly stressed fungal cells exhibit significantly promoted ability to survive when facing future insult^22,23^. In this study, secondary metabolic genes were found to be strongly affected in *A. flavus* under mild temperature stress and oxidative stress conditions. The co-regulation of different secondary metabolic pathways might contribute to maintaining cellular homeostasis and promoting cell survival under environmental stress.

Among the in vitro compounds affecting aflatoxin biosynthesis, ROS have been of particular interest because they are produced in response to biotic and abiotic stress and can act as signals for the regulation of fungal growth, development, and metabolism^5,9,24^. ROS and their reactive products, such as peroxidised lipids, have been shown to affect aflatoxin production^25,26^. However, because the function of aflatoxin biosynthesis in *A. flavus* biology is still unclear, studies on the relationship between ROS and aflatoxin production have led to different hypotheses. Previous studies have shown that aflatoxin biosynthesis in *A. flavus* is accompanied by increased oxygen consumption and is followed by a subsequent boost in ROS production^25^. The aflatoxisomes have also been demonstrated as one of the major locations for ROS production inside *A. flavus*^12,27^. The multiple cytochrome p450 monooxygenases and monooxygenases in the aflatoxin biosynthesis pathway are likely responsible for this burst in ROS generation. These results have led to the hypothesis that the aflatoxin biosynthetic pathway is a source of endogenous ROS production. In our study, the expression of aflatoxin biosynthetic genes was downregulated under oxidative stress, possibly as a mechanism to reduce endogenous ROS production, thus maintaining cellular ROS homeostasis. It has been suggested that the burst of ROS production during aflatoxin production may contribute to enhancing conidial oxidative tolerance in aflatoxigenic fungi^28^.

However, several previous studies have hypothesized that the opposite is true. Since aflatoxin biosynthesis has also been shown to be induced by oxidative stress, it has been suggested that aflatoxin biosynthetic pathway may supplement the antioxidation system in aflatoxigenic fungi, functioning to protect against ROS ^12,28,29^. Fountain et al. investigated the transcriptomic responses of different *A. flavus* isolates to H_2_O_2_-induced oxidative stress and found that aflatoxin biosynthetic genes were upregulated by oxidative stress in the moderate aflatoxin producer NRRL3357 but not in the high aflatoxin producer Tox4^30^. The authors suggest that differences in fungal growth and development among the different isolates under oxidative stress may have led to this. Aflatoxin and most of other secondary metabolites are produced after the fungus completes its initial growth phase and is beginning the stage of development that is characterized by sporulation^31,32^. Aflatoxin biosynthetic genes are typically expressed at higher levels during the aflatoxin production period, which is approximately 2 to 6 d in conducive media. In this study, stress conditions were applied to cultures after 2 d incubation, which is when aflatoxin production started, and total RNA samples were isolated after a short exposure time (24 h) in order to capture early and direct responses rather than profiles from a later stage, when both fungal growth and aflatoxin production have usually ceased, and fungal cells have adapted to the stress condition. Thus, the results in our study that differ from those of previous studies most likely reflect differences in the treatment periods examined.

In light of this, we hypothesize that aflatoxin production may serve different biological roles at different fungal growth stages. During early stages of fungal development, when sporulation has started, aflatoxin biosynthesis may function as a source of endogenous ROS production, which activates antioxidant enzyme expression and contributes to enhancing conidial oxidative tolerance; at the later stationary phase of growth, the aflatoxin biosynthetic pathway may instead function to fix excess oxygen into aflatoxin, which is then secreted from the fungal cells, thus providing a degree of oxidative tolerance in *A. flavus*.

Since fungal secondary metabolites are synthesized at their highest levels at specific time points in the fungal life cycle, it has been suggested that fungal cells possess complex molecular switches that regulate the activation of secondary metabolic pathway genes and the flow of primary metabolites through these pathways^17^. These molecular switches may also react to environmental stress and, in response, adjust the expression patterns of the genes involved in primary and secondary metabolism to enhance the survival of fungal cells. Among the secondary metabolic genes detected in our transcriptomic analysis, biosynthetic genes for kojic acid and imizoquins were differentially expressed. Kojic acid is a potent antioxidant that is synthesized from glucose that does not undergo glycolysis; it is also an iron-chelating antioxidant compound capable of scavenging ROS^33^. Imizoquins are a family of tripeptide-derived alkaloids produced via a nonribosomal peptide synthetase-derived tripeptide; they also protect against oxidative stress and have been shown to be essential for normal germination in *A. flavus*^34^. Under the oxidative stress condition, *A. flavus* may regulate the flow of primary metabolites, so that it can accumulate kojic acid and imizoquins to relieve the oxidative stress in its cells, thus resulting in fewer primary metabolites produced via the aflatoxin biosynthetic pathway. In fact, a negative correlation between aflatoxin and kojic acid biosynthesis has been reported^33^.

In this study, we found that the genes encoding mitochondrial complexes I and III were downregulated by temperature and oxidative stress. Under physiological conditions, endogenous ROS are generated mainly by the mitochondrial electron transport chain through these two respiratory chain components. Under stress conditions, besides depending on the antioxidation system, fungal cells may also downregulate cellular processes that generate ROS in order to avoid cellular oxidative damage. By contrast, the gene encoding mitochondrial complex IV was found to be upregulated. These different responses could be due to the fact that mitochondrial complex IV is not directly involved in ROS generation. Furthermore, when complex I and complex III were inhibited, the fungal cells may have promoted complex IV in order to maintain the cellular ATP supply. AOX is localized on the matrix side of the inner mitochondrial membrane, and it couples ubiquinol oxidation directly to the reduction of O_2_ to H_2_O, thus introducing a branch in the cytochrome-based electron transfer chain; this results in fewer protons migrating across the inner mitochondrial membrane, leading to lower ATP production through oxidative phosphorylation^35,36^. According to our results, the gene encoding AOX is upregulated under oxidative stress. The activity of AOX has been found to be associated with stress response, ROS control, cellular redox state, cellular energy demand, and metabolic homeostasis in different fungal cells^37,38^. As AOX is absent in mammals, it has also been investigated as a potential drug target for pathogenic fungi^39,40^.

AOX maintains mitochondrial respiration and is essential for metabolic functions in fungal cells when the classical electron transport chain is inhibited, maintaining fungal growth and viability. Meanwhile, secondary metabolism, such as aflatoxin biosynthesis, requires considerable amounts of ATP and NADPH^41^. In order to maintain cellular homeostasis during the aflatoxin production stage, fungal cells may rely on alternative respiratory pathways for reoxidation of NADH when oxidative phosphorylation is inhibited. Previous studies have found that aflatoxin biosynthesis implies a boost in oxygen uptake, which is followed by an increase of endogenous ROS production, and this occurs during the transition between trophophase and idiophase, when secondary metabolites begin to be abundantly produced^25^. AOX was also found to affect oxygen uptake in *Aspergillus* spp.^42,43^. Additionally, AOX plays a role in the production of sterigmatocystin, which is the penultimate intermediate in the biosynthesis of aflatoxin B1^42,44^. These observations clearly indicate that AOX plays important roles in the regulation of aflatoxin biosynthesis. Taken together, these facts suggest that AOX might be an effective target for the control of aflatoxin contamination. Further study will be required to elucidate the role of AOX in the regulation of secondary metabolism and the significance of this enzyme in antiaflatoxigenic practice.

In addition, it has been reported gene expression data has a low degree of correlation with proteomic data from studies on the traits involved in the response of *A. flavus* to environmental stress, which suggests that there is post-transcriptional regulation of protein accumulation in response to environmental stress in *A. flavus*^45,46^. The detection of DEGs involved in protein metabolism and modification and ribosome function may also be indicative of post-transcriptional regulation of temperature and oxidative stress response (Supplementary Fig. S2). For example, multiple genes encoding ribosomal structural constituents were found to be upregulated in *A. flavus* in response to temperature stress and oxidative stress, and DEGs involved in rRNA processing were also upregulated under oxidative stress. The dual stresses treatment resulted in more upregulated DEGs for tRNA processing and protein biosynthesis than either single-stress treatment. These results indicate the importance of post-transcriptional regulation at the protein level to the environmental stress response of *A. flavus* during its aflatoxin production stage.

**Figure 7:**
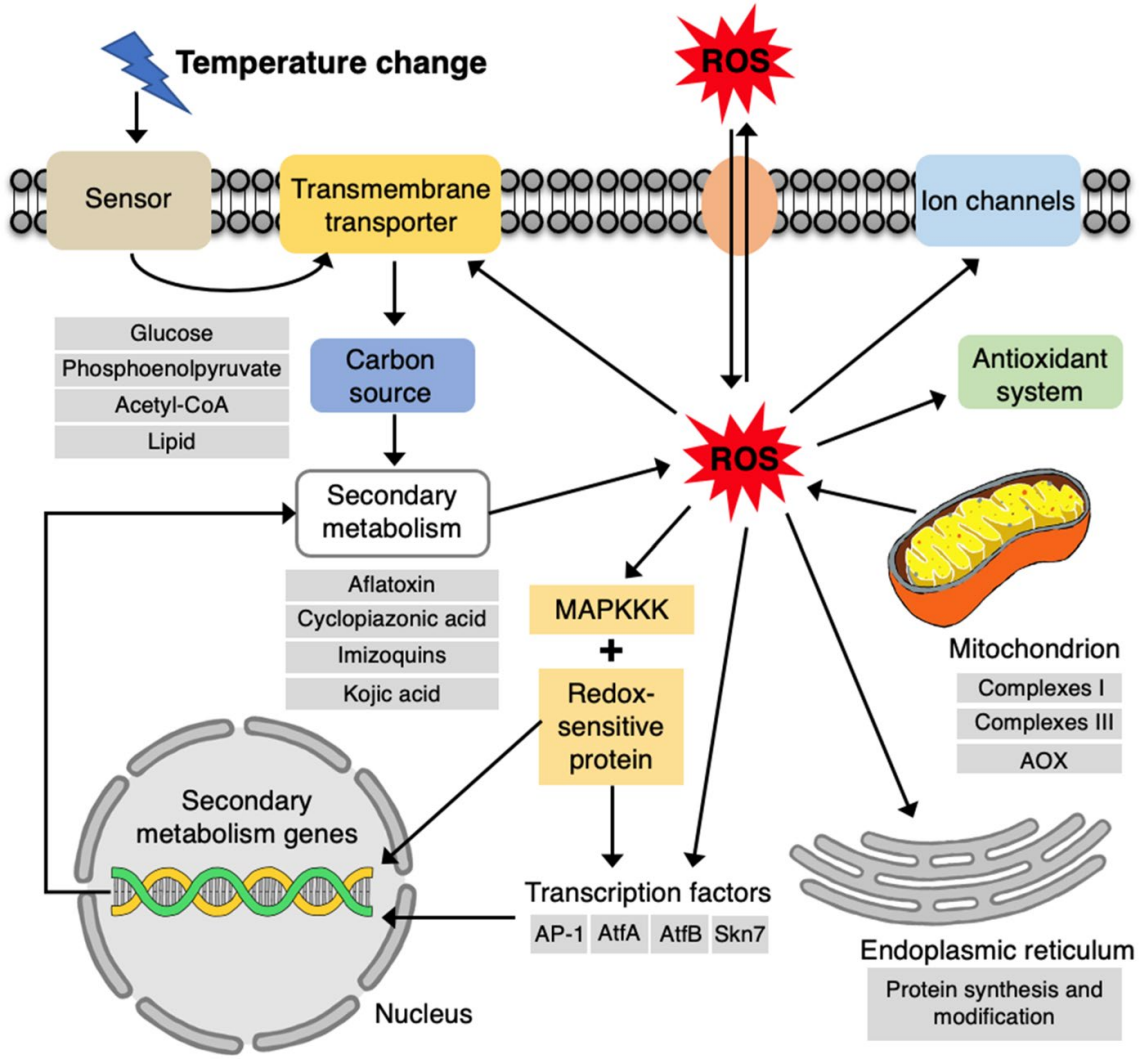
Overall response network exhibited by *A. flavus* to temperature and oxidative stress during aflatoxin production.

In summary, the transcriptomic responses of *A. flavus* to temperature stress and oxidative stress at the aflatoxin production stage revealed that secondary metabolic genes were strongly affected (Fig. 7). Aflatoxin biosynthetic genes were downregulated, while genes encoding secondary metabolites with antioxidative activity, such as kojic acid and imizoquins, were upregulated under stress conditions. The co-regulation of different secondary metabolic pathways in *A. flavus* might contribute to maintaining cellular homeostasis and promoting cell survival under environmental stress. Aflatoxin production may serve different biological functions at different fungal growth stages. During the early stages of fungal colonization, aflatoxin biosynthesis may function as a source of endogenous ROS production that activates antioxidant enzyme expression and enhances conidial oxidative tolerance. After reaching the stationary growth phase, the function of the aflatoxin biosynthetic pathway may be adjusted to instead fix excess oxygen into aflatoxin, which is then secreted from fungal cells, thus providing a degree of oxidative tolerance in *A. flavus*. Our results also highlight the important role played by mitochondria in fungal response to environmental stress. These results may provide a basis for identifying novel effective targets for controlling *A. flavus* and aflatoxin contamination under climate change.

## Methods

### Culture conditions

The *A. flavus* reference strain (KCCM 60330, also recorded as ATCC 22546) used in this study was collected from the Korea Culture Center of Microorganisms (KCCM, Seoul, Korea). Conidial suspensions for inoculation were prepared using fresh conidia harvested from potato dextrose agar (PDA) after 5 d incubation in the dark at 28 °C using 0.05% sterilized Tween 20 solution and diluted to the concentration of 10^6^ conidia/mL with autoclaved potato dextrose broth (PDB; Difco, BD, Franklin Lakes, NJ). Fungal incubation was conducted using a float culture method^18^, in which a polyvinylidene difluoride membrane (diameter = 47 mm, pore size = 0.45 μm, Merck, Darmstadt, Germany) was placed on the surface of autoclaved PDB in a 90-mm Petri dish. Fifteen microliters of fresh conidial suspension were inoculated onto the centre of the membrane. To test biomass and aflatoxin production, float culture plates were supplied with 25 mL of PDB and incubated for 1 to 7 d at a constant temperature of 28 °C. To test the transcriptomic responses of *A. flavus* to temperature stress and oxidative stress, each float culture plate was first supplied with 10 mL PDB. After 48 h of incubation at 28 °C, another 15 mL PDB with or without H_2_O_2_ (final concentration 5 mM) was added into each plate, which was then further incubated at 28 °C or 33 °C for 24 h. Two biological replicate cultures were performed for each treatment combination. All plates were incubated in the dark under stationary conditions.

### Biomass and aflatoxin production analysis

To analyse biomass and aflatoxin production in float cultures, membranes (carrying the fungal colonies) and culture broths were collected. Fungal biomass production was determined based on the dry weight of fungal colony on each membrane. Aflatoxin production was determined by a high-performance liquid chromatography system (Agilent 1200 LC system, Agilent Technologies, Santa Clara, CA, USA) using the previously described method^18^. Each test was conducted with three biological replicates for each sample and three technical replicates of each biological replicate.

### Total RNA extraction

After incubation, the whole fungal colony of *A. flavus* was removed from the membrane of each float culture and then immediately flash frozen in liquid nitrogen. Total RNA was then extracted from the mycelia using a QIAzol Lysis reagent kit (Qiagen, Hilden, Germany) according to the manufacturer’s instructions. The total RNA was then purified using a Riboclear plus kit (GeneAll, Korea). Quality and quantity of RNA samples were analysed using the Caliper LabChip GX system (Caliper Life Sciences, Hopkinton, MA, USA). RNA samples with quality scores more than 7 were used for transcriptome library construction and RNA sequencing.

### Transcriptome library construction and RNA sequencing

Transcriptome libraries were constructed using the NEXTflex Directional RNA-seq kit according to the manufacturer’s instructions (Bioo Scientific, Austin, TX, USA) with two biological replicates for each sample. Libraries were quantified using the KAPA qPCR kit (Kapa Biosystems, Woburn, MA). The libraries were sequenced on an Illumina HiSeq 2500 system to generate 151 bp × 2 paired-end reads for each sample at the National Instrumentation Center of Environmental Management, College of Agriculture and Life Science, Seoul National University (NICEM, Seoul, South Korea).

### Transcriptome assembly and bioinformatic analysis

Raw sequencing reads obtained from Illumina sequencing for each sample were first subjected to quality checking using FastQC (http://www.bioinformatics.bbsrc.ac.uk/projects/fastqc). Quality reads (> Q20) were trimmed to remove adaptor sequences and then used to perform genome-guided transcriptome assembly aligned to the *A. flavus* genome using Trinity assembler version 2.8.6. To remove redundancy, assembled contigs were clustered at 95% identity using CD-hit. Relative transcript abundances among the different samples were analysed in the cuffdiff program based on FPKM. DEGs were defined as genes with a log2 fold change > 0.5 and FDR < 0.05. Annotation of the assembled transcripts was performed by aligning their sequences against the uniprot database, the *A. flavus* coding sequences (CDS), and the NCBI non-redundant (nr) protein database with and e-value threshold of 10^–5^ using the BLASTx algorithm. All DEGs were annotated according to Gene Ontology (GO, http://geneontology.org/) databases to establish functional categories. *P*-values < 0.05 were considered significant.

### qRT-PCR analysis

In order to validate the gene expression differences detected by RNAseq, the expressions of 12 selected genes, involved in different bioactivities, were analysed using qRT-PCR with the previously described method^18^. In brief, RNA reverse transcription was conducted using a QuantiTect reverse transcription kit (Qiagen). A Bio-Rad CFX96 system (Bio-Rad, Hercules, CA, USA) was used to perform qRT-PCR. Gene expressions were tested using the Rotor-Gene SYBR green PCR kit (Qiagen) with specific primers (Supplementary Table S3), and analysed with CFX Manager software version 3.1 (Bio-Rad) using the ΔΔCT method^47^.

### Aflatoxin analysis

For the analysis of aflatoxin production, one milliliter of float culture was taken for the isolation of aflatoxin. Aflatoxin was extracted with the same volume of chloroform three times and cleaned with immunoaffinity columns (Vicam, Nixa, MO, USA), according to the manufacturer's instruction. The eluate was dried using N2 gas and then resuspended using 1 mL of 0.1% acetic acid–acetonitrile–methyl alcohol (59:14:29, v/v/v, mobile phase). Suspensions were filtered through a syringe filter (pore size, 0.2 μm) and stored at − 20 °C until analysis. Aflatoxin detection was performed using a high-performance liquid chromatograph (HPLC) system (Agilent 1200 LC system, Agilent Technologies, Santa Clara, CA, USA) with a reversed-phase C18 column (150 × 4.6 mm, 3.5 μm; Waters, Milford, MA, USA). The mobile phase was pumped for 30 min at a constant flow rate of 0.8 mL/min. Aflatoxin was detected in the 50 μL samples using a fluorescence detector (excitation at 360 nm, emission at 440 nm) and quantified based on a calibration curve of standard solutions of aflatoxin B1. The recovery rate of the chromatographic procedure for aflatoxin B1 was 85%, and the limit of detection (LOD) and limit of quantification (LOQ) were determined to be 0.036 and 0.109 ppb, respectively^18^.

## Supplementary Information

Supplementary Information.

Supplementary Data.

## Data Availability

All data generated or analysed during this study are included in this article and its Supplementary Information files. Sequencing data have been deposited in NCBI under Bioproject accession number PRJNA678857. RNA-seq raw data are accessible through Sequence Read Archive (SRA) accession number SRR13071529-44.
