# Author Correction: Transcriptomic responses of *Aspergillus flavus* to temperature and oxidative stresses during aflatoxin production

**DOI:** 10.1038/s41598-021-94955-2

**Published:** 2021-07-29

**Authors:** Fei Tian, Sang Yoo Lee, So Young Woo, Hwa Young Choi, Seongeun Heo, Gyoungju Nah, Hyang Sook Chun

**Affiliations:** 1 Food Toxicology Laboratory, School of Food Science and Technology, Chung-Ang University, Anseong, Korea; 2 Genome Analysis Center at National Instrumentation Center for Environmental Management, Seoul National University, Seoul, Korea

Correction to: *Scientific Reports* 10.1038/s41598-021-82488-7, published online 02 February 2021

The Acknowledgements section in the original version of this Article was incomplete.

“This work was supported by the Bio-Synergy Research Project of the Ministry of Science [Grant number NRF-2013M3A9C4078156], Republic of Korea.”

now reads:

“This work was supported by the Bio-Synergy Research Project of the Ministry of Science [Grant number NRF-2013M3A9C4078156] and a Chung-Ang University Graduate Research Scholarship in 2018, Republic of Korea."

The original Article has been corrected.

