# Supplementary material for: Transcriptomic responses of Aspergillus flavus to temperature and oxidative stresses during aflatoxin production
Source: Sci Rep. 2021 Feb 2;11:2803. doi: 10.1038/s41598-021-82488-7 (PMC7854668; doi:10.1038/s41598-021-82488-7)
Supplement: Supplementary file 1 — Supplementary Information. [file 41598_2021_82488_MOESM1_ESM.docx]

Transcriptomic responses of *Aspergillus flavus* to temperature and oxidative stresses during aflatoxin production

Fei Tian^1^, Sang Yoo Lee^1^, So Young Woo^1^, Hwa Young Choi^1^, Seongeun Heo^1^, Gyoungju Nah^2^, Hyang Sook Chun^1*^

^1^Food Toxicology Laboratory, School of Food Science and Technology, Chung-Ang University, Anseong, Korea. ^2^Genome Analysis Center at National Instrumentation Center for Environmental Management, Seoul National University, Seoul, Korea. ^*^Correspondence and requests for materials should be addressed to H.S.C. (email: hschun@cau.ac.kr)

Supplementary Table S1. Response of aflatoxin biosynthetic genes encoding different types of products (relative expression levels)

| Gene product type | Enzymes | Genes | Temperature  stress | Oxidative  stress | Dual stresses |
| --- | --- | --- | --- | --- | --- |
| Transmembrane transporter | transmembrane protein | *aflT* | 0.89 | 0.87 | 0.85 |
|  | putative hexose transporter | *aflYb/ hxtA* | 1.12 | 1.00 | 0.86 |
| Transcription regulator | transcription activator | *aflR* | 0.73 | 0.68 | 0.56 |
|  | pathway regulator | *aflS/aflJ* | 0.66 | 0.69 | 0.52 |
|  | sugar regulator | *aflYd/ sugR* | 0.72 | 0.89 | 0.73 |
| Reductase | fatty acid synthase | *aflA/fas-2/stcJ* | 0.68 | 0.59 | 0.36 |
|  | fatty acid synthase | *aflB/fas-1/stcK* | 0.62 | 0.55 | 0.35 |
|  | polyketide synthase | *aflC/pksA/stcA* | 0.63 | 0.52 | 0.31 |
|  | Reductase | *aflD/nor-1/stcE* | 0.55 | 0.58 | 0.31 |
|  | NOR reductase | *aflE/ norA* | 0.60 | 0.59 | 0.36 |
| Oxidoreductase | hypothetical protein | *aflY/hypA* | 0.59 | 0.67 | 0.33 |
|  | NADH oxidase | *aflYa/nadA* | 0.56 | 0.65 | 0.32 |
| O-methyltransferase | O-methyltransferase B | *aflO/omtB/stcP* | 0.60 | 0.66 | 0.37 |
|  | O-methyltransferase A | *aflP/omtA* | 0.65 | 0.61 | 0.37 |
| Monooxygenase | hypothetical protein | *aflCa/hypC* | 0.62 | 0.50 | 0.33 |
|  | monooxygenase | *aflN/verA/stcS* | 0.55 | 0.61 | 0.31 |
|  | monooxygenase | *aflW/moxY/stcW* | 0.64 | 0.63 | 0.32 |
|  | monooxygenase | *aflX/ordB/stcQ* | 0.59 | 0.58 | 0.39 |
| Hydrolyase | VERB synthase | *aflK/vbs/stcN* | 0.58 | 0.62 | 0.31 |
| Esterase | esterase | *aflJ/estA/stcI* | 0.58 | 0.55 | 0.33 |
| Dehydrogenase | dehydrogenase | *aflF/norB/stcV* | 0.99 | 1.00 | 0.75 |
|  | alcohol dehydrogenase | *aflH/adhA/stcG* | 0.50 | 0.60 | 0.33 |
|  | dehydrogenase | *aflM/ver-1/stcU* | 0.53 | 0.57 | 0.33 |
| Cytochrome P450 monooxygenase | cytochrome P450 monooxygenase | *aflG/avnA/stcF* | 0.56 | 0.62 | 0.29 |
|  | cytochrome P450 monooxygenase | *aflI/avfA/stcO* | 0.45 | 0.45 | 0.30 |
|  | P450 monooxygenase | *aflL/verB/stcL* | 0.68 | 0.64 | 0.37 |
|  | cytochrome P450 monooxygenase | *aflQ/ordA* | 0.59 | 0.66 | 0.37 |
|  | P450 monooxygenase | *aflU/cypA* | 1.18 | 0.90 | 0.82 |
|  | cytochrome P450 monooxygenase | *aflV/cypX/stcB* | 0.56 | 0.58 | 0.32 |

### Supplementary Table S2. Aflatoxin production of *A. flavus* under stress conditions

| **Treatment** | **Aflatoxin production**  **(μg/g mycelium)** |
| --- | --- |
| Control | 76.34 ± 5.53^a^ |
| Temperature stress | 35.73 ± 6.93^c^ |
| Oxidative stress | 57.64 ± 1.44^b^ |
| Dual stresses | 25.54 ± 3.38^d^ |

Values followed by different letters are significantly different according to Duncan's multiple range test at *P* < 0.05 using SPSS version 19 (IBM, Armonk, NY). All experiments were performed in triplicate.

Supplementary Table S3. List of qRT-PCR primers

| Gene name | Primers |
| --- | --- |
| *aflR* | F: 5’-GCACCCTGTCTTCCCTAACA-3’  R: 5’-ACGACCATGCTCAGCAAGTA-3’ |
| *aflS* | F: 5’-GGAATGGGATGGAGATG-3’  R: 5’-GGAATATGGCTGTAGGAAG-3’ |
| *aflD* | F: 5’-TCCAGGCACACATGATGGTC-3’  R: 5’-TGTGGATAACGAAGTGCCCC-3’ |
| *ap-1* | F: 5’-GGCCCATTGACCAAGTCAAACCAA-3’  R: 5’-CGGTTGTTTGAGCCGTTGAGTGTT-3’ |
| *atfA* | F: 5’-CACAGAACACGAACATGGATATG-3’  R: 5’-CTGA TTGGAAACAGCGAA TTG-3’ |
| *skn7* | F: 5’-CGGGCGAACAGCAAAGAATC-3’  R: 5’-TCAAACTGCCGAATGAGGTG-3’ |
| *aox1* | F: 5’-ACTTCCCAAGTGGACCAAGC-3’  R: 5’-CCCGTTGCTTTCAGATTCGC-3’ |
| *yat1* | F: 5’-CAGATGCGTTCGTGCAGATG-3’  R: 5’-TGCTGGATTCTCTCCCCAGA-3’ |
| *trx1* | F: 5’-CAGGAGAAGGTTATCGAATCCAAG-3’  R: 5’-GTCGACATCGATCTTGTAGAATTTGG-3’ |
| *mdr1* | F: 5’-TTTGTCGGGAGTCAGTGGTGTA-3’  R: 5’-CCAAGGGTTGTGCTAGTCATCA-3’ |
| *mfsC* | F: 5’-ATTGCCTCCATGGGAACAGG-3’  R: 5’-TGGAATACTGCTGGGCAAGG-3’ |
| *cyp51A* | F: 5’-GCCGGTCGAGCATTGC-3’  R: 5’-GCGAATTCTGCAGTGAGCTTT-3’ |
| *b-tubulin* | F: 5’-TCCAAGGTTTCCAGATCACC-3’  R: 5’-GAACTCCTCACGGATCTTGG-3’ |

Supplementary Figure S1. Relative expression and FPKM associations for select genes. qRT-PCR was used to determine the relative expression of 12 select genes: *aflR*, *aflS* and *aflD* (aflatoxin biosynthetic genes); *ap-1*, *atfA* and *skn7* (stress responsive transcription factors); *mdr1*, *mfsC* and *cyp51A* (drug resistance related genes); *aox1*, *yat1*, and *trx1* (mitochondria function related genes). The *A. flavus β-*tubulin gene was used as an internal control to normalize the expression data. The relative expression level of each gene was expressed as the fold change in the qRT-PCR data (blue bar) and the RNAseq data (orange bar). The bars represent the standard deviation from triplicates tests.

Supplementary Figure S2. Number of DEGs involved in protein metabolism and modification and ribosome function.
